# Successful Treatment of Human Visceral Leishmaniasis Restores Antigen-Specific IFN-γ, but not IL-10 Production

**DOI:** 10.1371/journal.pntd.0004468

**Published:** 2016-03-10

**Authors:** Emebet Adem, Fitsumbirhan Tajebe, Mulusew Getahun, Amare Kiflie, Ermias Diro, Asrat Hailu, Ziv Shkedy, Bewketu Mengesha, Tadele Mulaw, Saba Atnafu, Tekalign Deressa, Biniam Mathewos, Ebba Abate, Manuel Modolell, Markus Munder, Ingrid Müller, Yegnasew Takele, Pascale Kropf

**Affiliations:** 1 Department of Immunology, University of Gondar, Ethiopia; 2 Department of Internal Medicine, University of Gondar, Ethiopia; 3 Department of Microbiology, Immunology and Parasitology, Addis Ababa University, Ethiopia; 4 Department of Mathematics and Statistics, University of Hasselt, Belgium; 5 Leishmaniasis Research and Treatment Centre, Gondar University, Ethiopia; 6 Department of Cellular Immunology, Max-Planck-Institute for Immunobiology and Epigenetics, Freiburg, Germany; 7 Third Department of Medicine (Hematology, Oncology, and Pneumology), University Medical Center Mainz, Mainz, Germany; 8 Department of Medicine, Imperial College London, London, United Kingdom; Yale School of Public Health, UNITED STATES

## Abstract

One of the key immunological characteristics of active visceral leishmaniasis (VL) is a profound immunosuppression and impaired production of Interferon-γ (IFN-γ). However, recent studies from Bihar in India showed using a whole blood assay, that whole blood cells have maintained the capacity to produce IFN-γ. Here we tested the hypothesis that a population of low-density granulocytes (LDG) might contribute to T cell responses hyporesponsiveness via the release of arginase. Our results show that this population is affected by the anticoagulant used to collect blood: the frequency of LDGs is significantly lower when the blood is collected with heparin as compared to EDTA; however, the anticoagulant does not impact on the levels of arginase released. Next, we assessed the capacity of whole blood cells from patients with active VL to produce IFN-γ and IL-10 in response to antigen-specific and polyclonal activation. Our results show that whole blood cells produce low or levels below detection limit of IFN-γ and IL-10, however, after successful treatment of VL patients, these cells gradually regain their capacity to produce IFN-γ, but not IL-10, in response to activation. These results suggest that in contrast to VL patients from Bihar, India, whole blood cells from VL patients from Gondar, Ethiopia, have lost their ability to produce IFN-γ during active VL and that active disease is not associated with sustained levels of IL-10 production following stimulation.

## Author Summary

The leishmaniases, a group of diseases caused by *Leishmania* parasites, belong to the most neglected tropical diseases: they are mainly found in low-income countries and affect the poorest populations. These parasites infect cells of the immune system called macrophages, which can kill the intracellular parasites in response to soluble mediators they receive from other cells of the immune system, the lymphocytes. Visceral leishmaniasis is the most severe form of the leishmaniases and is characterized by enlarged liver and spleen, fever, weight-loss and anaemia and represents a major public health problem in Ethiopia. Currently there is no vaccine available, the existing treatment has many severe side effects and drug-resistance is increasing. In the present study, we worked with patients suffering from visceral leishmaniasis. This form of the disease is fatal if the patients are not treated. We studied the ability of lymphocytes isolated from their blood to produce soluble mediators before and at different times after the end of treatment. Our results show that the lymphocytes have an impaired capacity to produce the soluble mediator required to instruct infected cells to kill the intracellular parasites, but that this lack of response is gradually restored with time after successful treatment.

## Introduction

Visceral leishmaniasis (VL) is a neglected tropical disease caused by parasites of the *Leishmania (L*.*) donovani complex*. An estimated 200,000 to 400,000 new cases of VL with an incidence of 50,000 deaths occur each year, however these numbers are widely acknowledged to be a gross underestimation of the real burden [1,2]. In global estimates, Sudan, South Sudan, Ethiopia, Kenya and Somalia account for the second largest number of annual VL cases, after South Asia [1]

VL inflicts an immense toll on the developing world and impedes economic development, with an estimated loss of 2.3 million disability-adjusted life years. There is no effective vaccine; currently used chemotherapy is toxic and increasing drug resistance is reported [3]. VL can be asymptomatic or can manifest as a progressive disease characterised by hepatosplenomegaly, fever, weight loss, hyperglobulinemia and pancytopenia. In Ethiopia, VL is caused by *L*. *donovani* and it is one of the most significant vector-borne diseases; Ethiopia has the second largest number of VL cases in sub-Saharan Africa with an estimated annual burden of 4500 to 5000 new cases [2]. VL is worsened by malnutrition and HIV co-infection, and treatment access is often difficult because of the remote location of areas endemic for VL.

Non-healing VL in humans has been associated with increased levels of IL-10, a potent immunosuppressive cytokine (reviewed in [4]) and indeed, there is also ample evidence in the literature that patients with active VL are severely immunosuppressed and do not respond to the *Leishmanin* skin test. In addition, their PBMCs fail to produce IFN-γ and to proliferate in response to *Leishmania* antigen; this impaired capacity to respond to antigenic challenge is restored following successful chemotherapy [5] and reviewed in [4,6,7]. The mechanisms leading to these impaired T cell responses during symptomatic VL remain to be fully identified. We have recently shown that L-arginine depletion contributes to this lack of T cell responses: arginase-induced L-arginine metabolism has been identified as a potent mechanism of immune suppression [8–10]. We have shown previously in both experimental and human leishmaniasis that arginase activity is significantly increased in non-healing disease. In human leishmaniasis, we identified the phenotype of arginase-releasing cells as low-density granulocytes (LDGs), as these cells were collected in the PBMCs fraction, but not in the erythrocytes fraction. We have also established that LDGs are activated neutrophils that have degranulated and released arginase[11,12]. The subsequent elevated arginase in the microenvironment efficiently depletes L-arginine, an amino acid that is essential for efficient T cell responses, and this reduction in L-arginine results in impaired T cell responses [12,13].

Recent studies in Bihar, India, have challenged our current view on the apparent hyporesponiveness of PBMCs from patients with active VL to antigen-specific stimulation. Using a whole blood assay (WBA), the authors showed that whole blood cells produce IFN-γ in response to antigenic activation [14,15] and identified CD4^+^ T cells as the main type of IFN-γ producing cells [16]. The levels of IFN-γ were similar before and after successful treatment, suggesting that the inability of these patients to control the disease was not due to a defect in Th1 responses. In contrast, IL-10 production was elevated in the group with active VL and significantly reduced in cured patients.

In the present study, we evaluated the responsiveness of whole blood cells from a cohort of patients with active VL in Gondar, North West of Ethiopia. We first assessed whether the frequency of immunomodulatory LDGs in the blood of patients with active VL was affecting the levels of arginase activity in a WBA and might therefore affect the levels of cytokines. In the next step, we evaluated the antigen-specific production of IFN-γ and IL-10 over time in these patients.

## Materials and Methods

### Ethics statement

This experimental study was approved by the Institutional Review Board of the University of Gondar (IRB, reference SBMLS/1199/07) and informed written consent was obtained from each patient and control.

### Subjects and sample collection

For this cross-sectional study, a cohort of 23 patients with active visceral leishmaniasis (VL patients), whose diagnosis of VL was based on positive serology (rK39) and presence of amastigotes in spleen or bone marrow aspirates [17] was recruited from the Leishmaniasis Treatment and Research Center of Gondar University Hospital before treatment. All the patients in this study presented with fever, hepatosplenomegaly, pancytopenia and low BMI. Their age, duration of illness, parasite grade and treatment are summarized in Table 1. In addition, 16 VL patients after the end of successful treatment (17 days = TOC (Test Of Cure)), 20 patients 3 months following successful treatment; 10 patients 6 months following successful treatment (from different groups of patients at each time point); and 10 non-endemic healthy age- and sex-matched individuals (controls) coming from the city of Gondar, which is non-endemic for visceral leishmaniasis were recruited at the Leishmaniasis Treatment and Research Center of Gondar University Hospital. TOC was defined as follows: at the end of successful treatment, patients look improved, afebrile, and usually have a smaller spleen size than on admission and have an increased haemoglobin (Hgb) level.

**Table 1:** Clinical data.

	Age	Duration of illness(weeks)	Parasite grade(spleen)	Parasite grade(bone marrow)	Treatment
**VL 1**	42	24	1		Ambisome
**VL 2**	29	4	3		SSG+PM
**VL 3**	22	3	3		SSG+PM
**VL 4**	18	12	5		SSG+PM
**VL 5**	22	12	3		SSG+PM
**VL 6**	17	4		1	Ambisome
**VL 7**	28	8	2		SSG+PM
**VL 8**	33	8	4		SSG+PM
**VL 9**	45	4		1	SSG+PM
**VL 10**	25	3	5		SSG+PM
**VL 11**	31	4	3		SSG+PM
**VL 12**	28	8	5		Ambisome
**VL 13**	30	8	2		SSG+PM
**VL 14**	24	4	2		SSG+PM
**VL 15**	32	4	2		SSG+PM
**VL 16**	22	16	3		SSG+PM
**VL 17**	23	8	3		SSG+PM
**VL 18**	18	6	4		Ambisome
**VL 19**	30	2	4		SSG+PM
**VL 20**	21	12	2		SSG+PM
**VL 21**	18	20		2	SSG+PM
**VL 22**	20	12	1		SSG+PM
**VL 23**	28	12		1	SSG+PM

Age, duration of illness (defined as the number of weeks since the patients noticed symptoms associated with visceral leishmaniasis, such as fever, and/or enlarged abdomen (as a sign of enlarged spleen or liver)), parasite burden was graded as follows: 1 = 1–10 parasites/1000 fields, 2 = 1–10 parasites/100 fields, 3 = 1–10 parasites/10 fields, 4 = 1–10 parasites/ field, 5 = 10–100 parasites/field, 6 = >100 parasites/field [17].

No women presented with visceral leishmaniasis during our study, all patients were male migrant workers. Patients < 18 years old or presenting with tuberculosis or malaria were excluded from the study. All VL patients were routinely screened for HIV using the following tests: KHB Shanghai Kehua Bio-engineering Co. Ltd and Chembio HIV 1/ 2 STAT-PAK; Uni-Gold (Trinity Biotech PLC) was used to resolve ambiguous results; all patients enrolled in our study were HIV negative.

4–8 ml of blood was collected in EDTA and/or heparin tubes. Patients were treated with a combination of sodium stibogluconate (SSG, 20mg/kg body weight/day), and paromomycin (PM, 15mg/kg body weight/day) injections, given intramuscularly for 17 days or with Ambisome (max of 30mg/kg body weight, with 6 injections of 5mg/kg body weight /day) and showed an initial clinical cure rate of 100% after treatment (TOC).

### Flow cytometry

Antibodies used were as follows: anti-CD15 (Clone H198, BD Pharmingen), anti-arginase I (HyCult Biotechnology: clone 6G3) and the isotype control (BD Pharmingen: clone MOPC21) coupled with Alexa Fluor 647 (Molecular Probes). Cells were washed with PBS, the fixation step was performed with 2% formaldehyde in PBS and the permeabilization step with 0.5% saponin in PBS.

The determination of intracellular arginase was performed as described in [18]. The percentages for the isotype controls were <1%. Acquisition was performed using a FACSCalibur (BD Biosciences) and data were analyzed using Summit v4.3 software.

### Determination of arginase activity

Arginase activity was measured as described in [18]. To determine arginase activity in plasma from stimulated blood samples, urea concentrations were first determined without the activation and hydrolysis steps; these values were subtracted from those obtained by measuring the urea levels as described in [11]. One unit of enzyme activity is defined as the amount of enzyme that catalyzes the formation of 1 μmol of urea per min.

### Collection of blood and stimulation of cells

Three x 2ml of blood were collected in EDTA tubes and 3 x 2ml in heparin tubes (BD). Soluble *Leishmania* antigen (SLA) was prepared from stationary-phase *L*. *donovani* promastigotes isolated from 5 patients as described in [14], and was added immediately after blood collection at a concentration of 5μg/mL and phytohaemagglutinin (PHA, Sigma) at 10 μg/mL. For the stimulation in the presence of L-arginine, two x 2ml tubes of blood were collected and 1mM L-arginine (Sigma) was added directly in the tubes. Unstimulated blood was used as negative control (nil). Plasma from activated blood samples and negative controls was collected after 24 hours of incubation at 37°C and stored at -20°C for further analysis.

### Measurement of interferon-gamma and interleukin-10 by ELISA

The level of IFN-γ and IL-10 in the plasma from stimulated blood was measured using Human IFN-γ and IL-10 ELISA Ready-SET-Go! kit using the manufacturer’s instructions and procedure (eBioscience). Antigen-specific IFN-γ and IL-10 levels (expressed in pg/mL) produced in response to SLA and PHA stimulation were determined by subtracting background levels measured in the non-stimulated samples (nil). The detection limit for IFN-γ and IL-10 was 2 pg/ml.

### Statistical analyses

Data were analyzed for statistical differences using nonparametric two-sided Mann-Whitney, Wilcoxon or Kruskal-Wallis tests (GraphPad Prism 6) when appropriate and differences were considered statistically significant at *p* < 0.05. The Bonferroni method was used for multiplicity correction whenever it was needed. Unless otherwise specified, results are expressed as median ± SEM.

## Results

### Impact of the anti-coagulant on the frequency of low-density granulocytes

**Fig 1:**
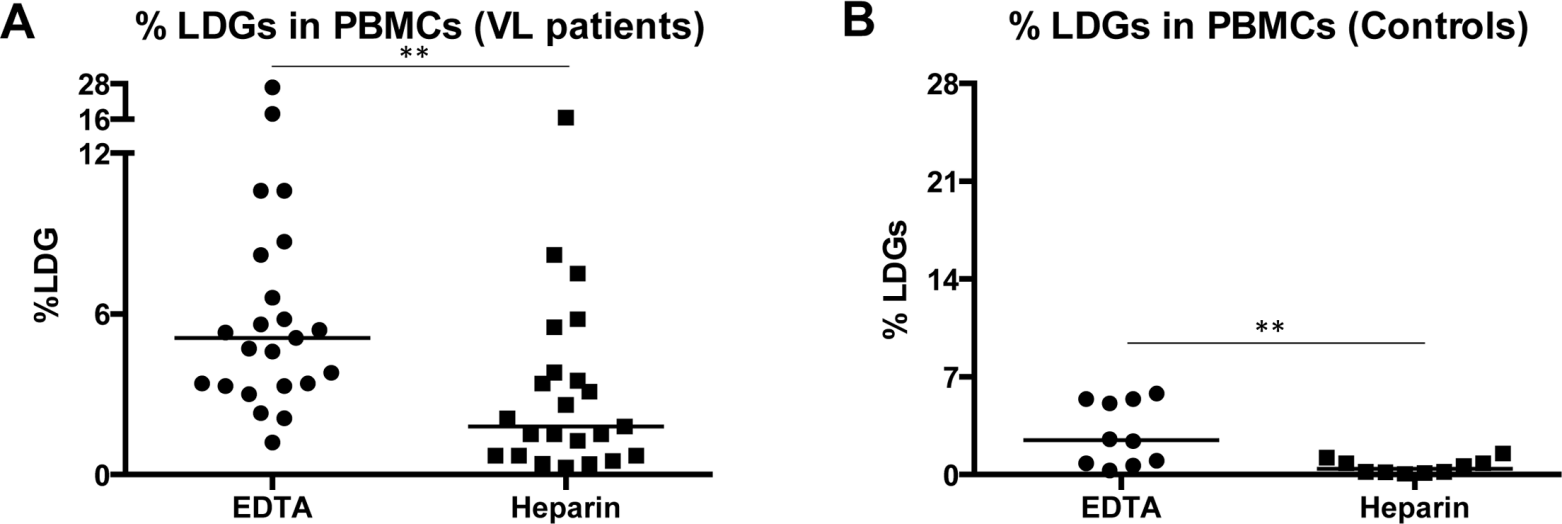
Frequency of LDGs in blood collected with EDTA and heparin. Blood from VL patients (n = 23) and non-endemic healthy controls (n = 10) was collected with heparin and EDTA, PBMCs were isolated by Ficoll gradient and the frequencies of LDGS (= CD15+ arginase+ cells[12]) were determined by flow cytometry. Each symbol represents the value for one individual, the straight lines represent the median, and statistical differences were determined using a Mann-Whitney test. A) VL patients, B) controls.

We have previously shown that the frequency of LDGs is significantly increased in patients with active VL [12]. Since these cells have been shown to have immunomodulatory properties, we proposed that LDG-mediated T cell suppression is a key element in the outcome of VL. Our preliminary data also suggested that the anticoagulant used to collect blood had a remarkable effect on the survival of LDGs *ex vivo* [12]. Here, we tested the impact of two commonly used anticoagulants, EDTA and heparin, on the frequencies of LDGs: blood was collected from VL patients in EDTA and heparin tubes, their PBMCs were isolated by Ficoll gradient and the frequencies of CD15^+^arginase^+^ cells (LDGs [12]) were determined by flow cytometry. Results presented in Fig 1 show that the frequency of LDGs is significantly lower in PBMCs isolated from blood from VL patients collected in heparin as compared to EDTA tubes (Fig 1A, Table 2). Similar results were obtained with blood from controls collected in EDTA and heparin (Fig 1B, Table 2), indicating that heparin affects the frequency of LDGs not only in patients with active VL but also in controls.

**Table 2:** Frequency of LDGs.

	EDTA	Heparin	*p* values Mann-Whitney
VL patients	5.10 ± 1.91	1.80 ± 0.77	*p* = 0.0013
Controls	2.47 ± 0.72	0.40 ± 0.16	*p* = 0.0048

Blood from VL patients (n = 23) and non-endemic healthy controls (n = 10) was collected with heparin and EDTA, PBMCs were isolated by Ficoll gradient and the frequencies of CD15+ arginase+ cells were determined by flow cytometry. Statistical differences between the frequencies of LDGs in the blood collected on EDTA and heparin were determined using a Mann-Whitney test. Results are expressed as median ± SEM.

### Impact of EDTA and Heparin on cytokine production and arginase release in the whole blood assay

It has been reported recently that whole blood cells from patients with active VL maintain the capacity to produce IFN-γ and IL-10 following activation of whole blood with soluble *Leishmania* antigen (SLA) [15]. Here we first tested the impact of heparin and EDTA on the production of IFN-γ in the whole blood assay (WBA). To obtain the levels of IFN-γ produced following stimulation of whole blood cells with SLA or PHA, the background levels measured in the non-stimulated samples (nil) were substracted from the activated samples. The production of IFN-γ in the WBA in response to SLA was low or below detection limit, independently of the anticoagulant used (EDTA: 54.1±23.9 vs heparin: 61.3±54.0, *p* = 0.3125, data not illustrated). Similar results were obtained following polyclonal activation with PHA (EDTA: 42.9±34.8 vs heparin: 41.8±19.9, *p* = 0.6523, data not illustrated).

**Fig 2:**
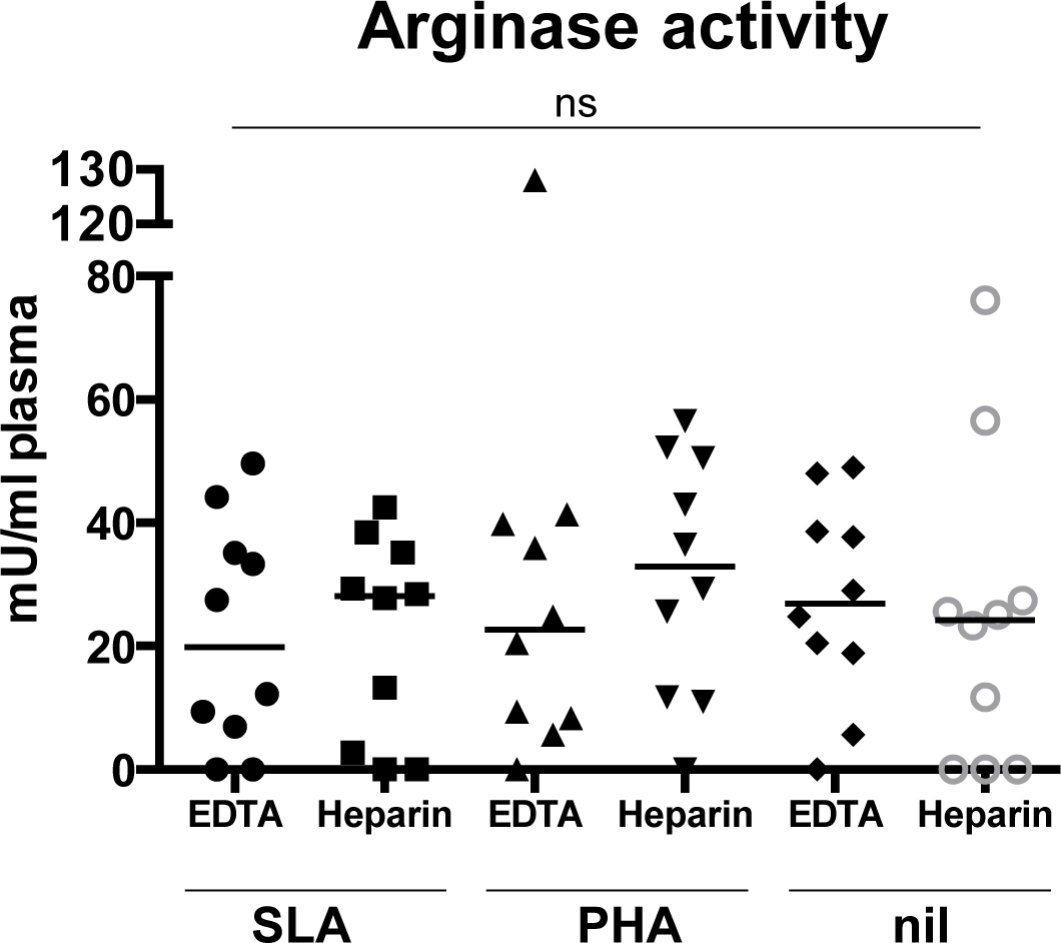
Arginase activity in plasma from activated whole blood cells. Blood from VL patients (n = 10) was collected with heparin and EDTA and was activated at 37°C with SLA, PHA or PBS (unstimulated). 24 hours later, the plasma was collected and the levels of arginase activity were measured by enzymatic assay as described in materials and methods. Each symbol represents the value for one individual, the straight lines represent the median. Statistical differences were determined using a Kruskal-Wallis test.

We have previously shown that arginase-induced L-arginine depletion can suppress T cell activation and cytokine release [13]. To determine whether the poor IFN-γ response observed in the WBA could be due to increased levels of arginase activity released in the microenvironment, we measured the activity of arginase in the plasma after the 24 hours incubation. Results presented in Fig 2 show that the levels of arginase activity were not significantly affected by the anticoagulant used (summarized in Table 3). The levels of arginase activity were similar in all groups tested (Fig 2, *p* = 0.949), suggesting that the anticoagulants used did not impact on the production of IFN-γ via increased release of arginase.

**Table 3:** Arginase Activity in plasma from whole blood assay.

	EDTA	Heparin	*p* values
SLA	19.85 ± 5.81	28.10 ± 5.17	*p* = 0.9102
PHA	22.65 ± 11.72	32.90 ± 6.17	*p* = 0.4316
nil	26.90 ± 5.25	24.20 ± 7.93	*p* = 0.8457

Blood from VL patients (n = 10) was collected with heparin and EDTA and was activated at 37°C with SLA, PHA or PBS (nil). 24 hours later, the plasma was collected and the activity of arginase was determined by enzymatic assay as described in materials and methods. Statistical differences between arginase activity in the plasma of the WBA collected on EDTA and heparin were determined using a Mann-Whitney test. Results are expressed as median ± SEM.

**Fig 3:**
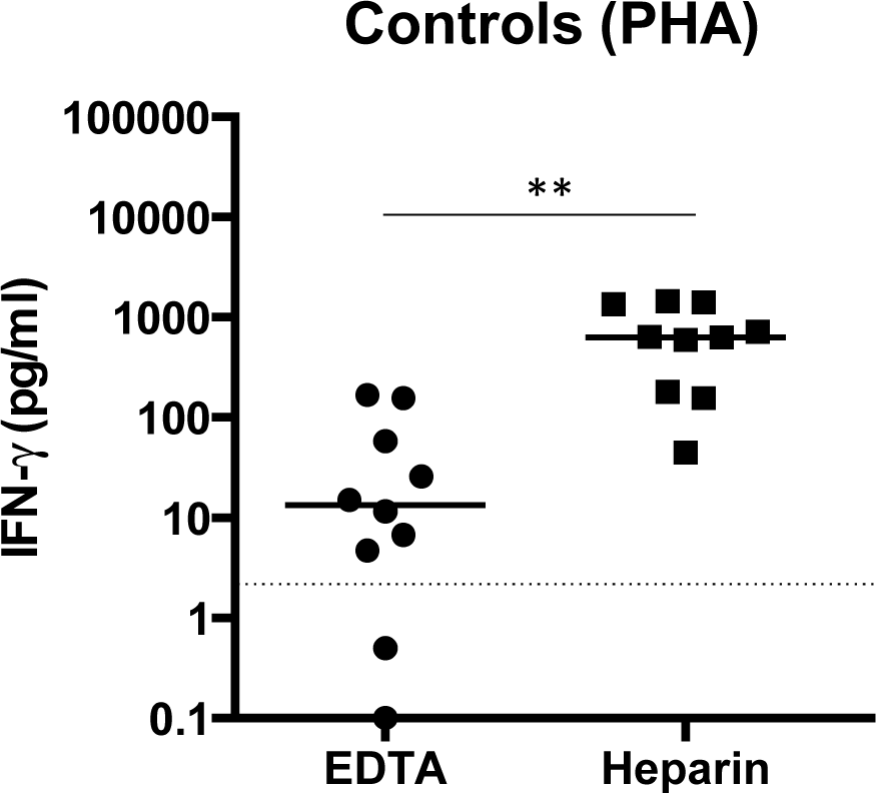
IFN-γ levels in plasma from activated whole blood cells. Blood from non-endemic healthy controls (n = 10) was collected with heparin and EDTA and was activated at 37°C with PHA or PBS (nil = unstimulated). 24 hours later, the plasma was collected and the levels of IFN-γ were measured by ELISA as described in materials and methods and in exactly the same conditions and in the same laboratory as the VL patients. The value obtained for the unstimulated cells (nil) was subtracted from the values obtained for PHA stimulations. Each symbol represents the value for one individual, the straight lines represent the median, and statistical differences were determined using a Mann-Whitney test. The dotted line represents the detection limit.

Our results show that the IFN-γ production is low or below detection limit in the WBA and that this is unlikely to be due to arginase-induced L-arginine depletion. To exclude any technical problem with the IFN-γ ELISA assay, we stimulated whole blood cells from non-endemic controls with PHA in exactly the same conditions and in the same laboratory as the VL patients. Results presented in Fig 3 show that following polyclonal activation, IFN-γ was clearly detectable when the blood was collected with heparin, indicating that the unresponsiveness of some VL patients to PHA in the WBA is not due to technical issues. Since EDTA chelates the Ca^+^ needed for cellular activation, IFN-γ was low or below detection limit when the blood was collected with EDTA (630.4±166.1 vs 13.3±20.0, *p* = 0.0020, Fig 3). As expected [19], these results show that collecting blood in EDTA prevents the production of IFN-γ. IFN-γ was low or below detection limit in the supernatant of whole blood from non-endemic healthy controls activated with SLA (2.10 ±1.19 pg/ml, data not illustrated).

### Comparison of IFN-γ and IL-10 production by stimulated whole blood cells from patients with active VL and cured patients

**Fig 4:**
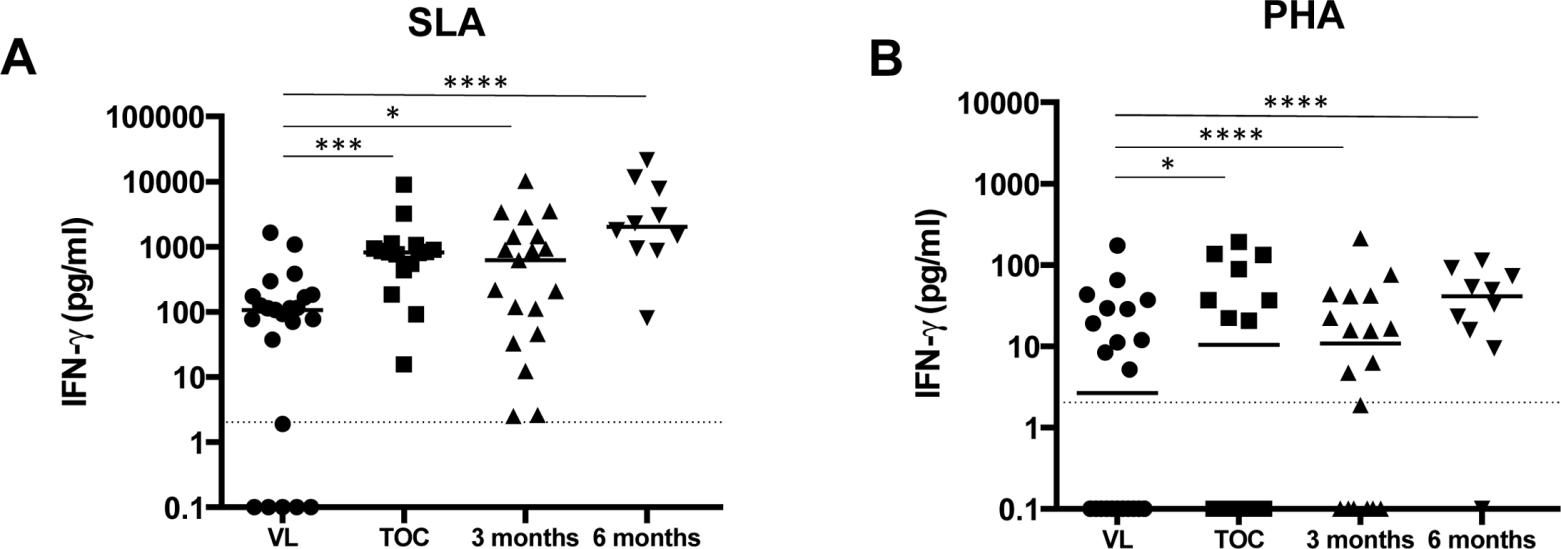
Comparison of IFN-γ production in patients with active VL and in cured patients. Blood from patients with active VL (VL, n = 23), after 17 days (TOC, n = 16), 3 months (n = 20) or 6 months (n = 10) of treatment was collected with heparin and was activated at 37°C with SLA, PHA or PBS (nil). 24 hours later, the plasma was collected and the levels of IFN-γ were measured by ELISA as described in materials and methods. The value obtained for the unstimulated cells (nil) was subtracted from the values obtained for SLA (A) and PHA (B) stimulations. Each symbol represents the value for one individual, the straight lines represent the median and statistical differences were determined using a Mann-Whitney test. The dotted line represents the detection limit.

IFN-γ is elevated in the plasma of VL patients [4] and indeed our data show elevated levels of IFN-γ in plasma from active VL patients as compared to controls (123.0±27.5 pg/ml vs 31.1±23.2pg/ml, *p* = 0.0030, Table 4). Recent studies in Bihar, India, have shown that the production of antigen-specific IFN-γ in the WBA from patients with active VL was similar to that in cured VL patients, suggesting that T cells from these patients have the capacity to respond to antigenic challenge by producing significant amounts of IFN-γ [15]. These results were contrary to previous studies showing that PBMCs from VL patients do not produce IFN-γ after stimulation with *Leishmania* antigen (reviewed in [4,6]). To evaluate the capacity of whole blood cells from VL patients in Gondar, Ethiopia, to respond to antigenic challenge over time by using the WBA, we performed a cross-sectional study and collected blood in heparin before the start of the treatment, at the end of successful treatment (for 17 days, TOC) and 3 and 6 months after treatment (from different groups of patients at each time point) and activated blood cells with SLA and PHA. In contrast to the results obtained previously in India, the levels of antigen-specific IFN-γ were increased significantly and gradually after successful treatment (for 17 days, TOC), 3 months and finally 6 months (Fig 4A, Table 5). Similar results were obtained in response to polyclonal activation (Fig 4B, Table 5): the production of IFN-γ in response to PHA was low or below detection levels before treatment. To further determine whether the lack of clear response was due to low levels of L-arginine in the plasma of these patients [12], whole blood from active VL patients was activated with PHA in the presence or absence of L-arginine and the resulting levels of IFN-γ produced were similar in both groups (123.9±84.2 vs 119.8±42.9, respectively, *p* = 0.6667, data not illustrated).

**Table 4:** IFN-γ and IL-10 levels in plasma of VL patients and controls.

	IFN-γ	IL-10
VL	123.0±27.5	88.9±12.5
Controls	31.1±23.2	4.0±5.5
*p* value	0.0030	0.002

Plasma from patients with active VL (VL, n = 29) and healthy controls (controls, n = 14) were collected and the levels of IFN-γ and IL-10 were measured by ELISA as described in materials and methods. Statistical differences were determined using a Mann-Whitney test. Results are expressed as median ± SEM.

**Table 5:** IFN-γ production in plasma from whole blood assay.

	SLA	*p* values (vs VL)	PHA	*p* values (vs VL)
VL	107.0±80.6		21.4±88.4	
TOC	824.6±541.1	*p* = 0.0001	139.8±164.4	*p* = 0.0276
3 months	624.7±557.3	*p* = 0.0130	798.1±215.2	*p*<0.0001
6 months	2040.0±2145.0	*p*<0.0001	996.9±943.1	*p*<0.0001

Blood from patients with active VL (VL), after 17 days (TOC), 3 months or 6 months of treatment was collected with heparin and was activated at 37°C with SLA, PHA or PBS (nil). 24 hours later, the plasma was collected and the level of IFN-γ was measured by ELISA as described in materials and methods. Statistical differences were determined using a Mann-Whitney test and using the Bonfferoni method for multiplicity correction (i.e. p values are compared with 0.0166 = 0.05/3). Results are expressed as median ± SEM.

**Fig 5:**
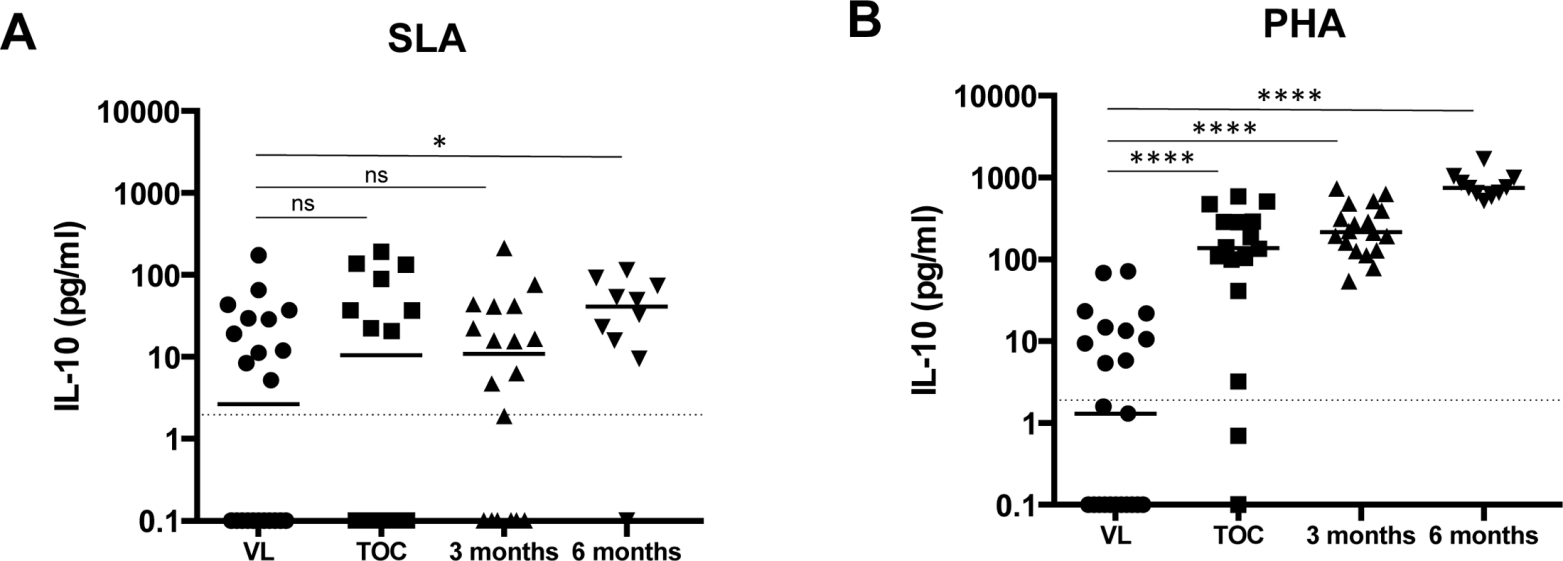
Comparison of IL-10 production in patients with active VL and in cured patients. Blood from patients with active VL (VL, n = 23), after 17 days (TOC, n = 16), 3 months (n = 20) or 6 months (n = 10) of treatment was collected with heparin and was activated at 37°C with SLA, PHA or PBS (nil). 24 hours later, the plasma was collected and the level of IL-10 was measured by ELISA as described in materials and methods. The value obtained for the unstimulated cells (nil) was subtracted from the values obtained for SLA (A) and PHA (B) stimulations. Each symbol represents the value for one individual, the straight lines represent the median and statistical differences were determined using a Mann-Whitney test. The dotted line represents the detection limit.

In the study conducted in India, IFN-γ levels detected in the WBA were similar before and after treatment, in contrast, IL-10 levels were elevated during active VL, but reduced significantly in cured patients, suggesting that IL-10 is associated with active disease [15]. Furthermore, IL-10 is also elevated in the plasma from patients with active VL, and our results also show that plasma IL-10 is significantly increased in VL as compared to healthy controls (88.9±12.5 vs 4.0±5.5pg/ml, respectively, *p* = 0.0002, Table 4). Here we compared the levels of IL-10 in the plasma of the WBA over time. Unexpectedly, results presented in Fig 5A show that antigen-specific IL-10 levels are low or below detection limit during active VL, after the 17 days of treatment (TOC) and 3 and 6 months after the end of successful treatment (summarized in Table 6). These results suggest that active VL is not associated with high IL-10 production in the WBA in the cohort of VL patients from North West Ethiopia. However, blood cells from these patients have the capacity to produce IL-10 in response to polyclonal activation after treatment, as results in Fig 5B show gradually increasing levels of IL-10 after successful treatment (for 17 days, TOC), 3 months and finally 6 months (Fig 5B, Table 6).

**Table 6:** IL-10 production in plasma from whole blood assay.

	SLA	*p* values (vs VL)	PHA	*p* values (vs VL)
VL	2.6±8.3		1.3±4.2	
TOC	10.4±15.3	*p* = 0.5059	136.4±46.6	*p*<0.0001
3 months	10.9±12.0	*p* = 0.3918	215.1±45.8	*p*<0.0001
6 months	41.2±11.8	*p* = 0.0103	749.2±106.8	*p*<0.0001

Blood from patients with active VL (VL), after 17 days (TOC), 3 months or 6 months of treatment was collected with heparin and was activated at 37°C with SLA, PHA or PBS (nil). 24 hours later, the plasma was collected and the level of IL-10 was measured by ELISA as described in materials and methods. Statistical differences were determined using a Mann-Whitney test and using the Bonfferoni method for multiplicity correction (i.e. p values are compared with 0.0166 = 0.05/3). Results are expressed as median ± SEM.

Our results presented in Table 7 also show that whole blood cells from VL patients cannot be induced to secrete these cytokines *in vitro* since the levels of both cytokines in response to SLA and nil or PHA and nil were similar in the plasma of the WBA. However, significant differences in IFN-γ levels between SLA and nil and PHA and nil were observed after successful treatment (for 17 days, TOC), 3 and 6 months after the end of treatment, demonstrating that in this setting, production of IFN-γ can be induced after successful treatment, but not at time of active VL. A similar conclusion can be made with the production of IL-10 in response to PHA.

**Table 7:** Comparison of unstimulated versus stimulated cultures.

IFN-γ	SLA	nil	*p* value	PHA	nil	*p* value
**VL**	287.2±137.8	165.8±148.8	0.0783	171.8±141	165.8±148.8	0.6445
**TOC**	915.3±189.0	6.4±85.9	<0.0001	209.3±91.6	6.4±85.9	0.0108
**3 months**	929.9±301.0	0.1±79.0	<0.0001	936.5±152.8	0.1±79.0	<0.0001
**6 months**	1124.1±845.3	15.5±5.8	<0.0001	1115.0±719	15.5±5.8	<0.0001
**IL-10**						
**VL**	21.5±25.4	35.0±10.5	0.5240	48.4±9.4	35.0±10.5	0.7565
**TOC**	71.3±20.2	57.7±19.8	0.6494	208.7±57.7	57.7±19.8	0.0030
**3 months**	44.8±13.4	21.3±11.7	0.1732	286.4±46.2	21.3±11.7	<0.0001
**6 months**	70.3±8.1	6.3±11.2	0.0157	752.7±105.7	6.3±11.2	<0.0001

Blood from patients with active VL (VL), after 17 days (TOC), 3 months or 6 months of treatment was collected with heparin and was activated at 37°C with SLA, PHA or PBS (nil). 24 hours later, the plasma was collected and the levels of IFN-γ and IL-10 were measured in the plasma from whole blood cells cultured in the presence of SLA, PHA and nil, by ELISA as described in materials and methods. Statistical differences were determined using a paired t test. Results are expressed as median ± SEM.

These results demonstrate that in patients from Gondar, whole blood cells are hyporesponsive during the active phase of the disease, however, this is progressively reversed after successful treatment. Furthermore, our results show that increased IL-10 production by whole blood cells is not a hallmark of active VL in patients from Gondar.

## Discussion

Recently, the existing dogma on T cell hyporesponsiveness during active VL was challenged by data showing that VL patients from India maintain the capacity to produce IFN-γ in the WBA [15]; these studies suggest that the inability of these patients to control the disease was not due to a defect in the Th1 response. We first tested whether the anticoagulant used in the WBA might impact on the frequency of LDGs, a subpopulation of neutrophils with immunomodulatory properties that can suppress the production of cytokines. Indeed, our preliminary data suggested that the use of heparin, the anticoagulant used in the WBA discussed above, results in a sharp reduction of the frequency of LDGs [20]. Our results show that the frequency of LDGs is drastically reduced when the blood is collected in heparin, suggesting that the use of heparin as anticoagulant could result in underestimated frequencies of LDGs. Whereas EDTA has been shown to impact on biological functions of neutrophils [21], their viability does not seem to be affected by different anticoagulants [21,22]. EDTA chelates the free calcium needed as a cofactor to activate enzymes responsible for coagulation, whereas heparin blocks coagulation by activating antithrombin. LDGs are a distinct subpopulation of highly activated neutrophils; thus, it is possible that LDGs isolated from blood harvested in EDTA lack Ca^+^ required to undergo cell death and therefore survive longer in EDTA than LDGs harvested in the presence of heparin. Similarly, the chelation of Ca+ by EDTA explains the lack of IFN-γ production in whole blood assays [19].

We cannot formally exclude the possibility that EDTA may results in activation and thereby degranulation of neutrophils, however, we and others have shown that the frequency LDGs is increased in several conditions, such as HIV[23], SLE [24], visceral leishmaniasis[12], pregnancy[18], asthma[25] and cancer[26], supporting our conclusions that this is specific to inflammatory conditions in the affected individuals. Furthermore, our samples are processed immediately and it is therefore unlikely that neutrophils from VL patients are "more" activated then those from controls in this short period of time.

Release of arginase in the microenvironment results in the depletion of extracellular L-arginine, that in turn prevents T cell activation [10]. We have previously shown that in patients with active VL, the frequency of activated LDGs in their PBMCs is significantly increased, that these cells express significantly less intracellular arginase and that the levels of arginase in the plasma is significantly increased [12]. We considered the possibility that the lack of IFN-γ response in the WBA could be due to increased levels of released arginase: however, our results show that the levels of arginase activity are similar in all plasma samples harvested 24 hours after activation of whole blood cells showing that enhanced arginase release is not accounting for the hyporesponsiveness of the cells in the WBA. But we cannot exclude that LDGs might still account for the observed hyporesponsiveness via other mechanisms such as cell-cell contact or release of molecules. However, we can conclude from our results that cells from whole blood cells collected from patients with active VL produce low or no IFN-γ in response to antigenic or polyclonal activation, suggesting that blood cells from patients with active VL are hyporesponsive. We can exclude technical problems with our assay, as IFN-γ was clearly detectable in the supernatant of whole blood cells from healthy controls activated with PHA. Our results are in agreement with ample evidence from the literature showing that one of the key immunological characteristics of active VL is profound immunosuppression, as demonstrated by the failure of PBMCs to produce IFN-γ and proliferate in response to *Leishmania* antigen (reviewed in [4,6]). Whereas *ex vivo*, PBMCs from patients with active VL cannot be induced to produce IFN-γ and IL-10 in response to antigenic or polyclonal stimulation, it is still possible that responsive cells remain at the site of pathology; indeed, IFN-γ and TNF-α have been detected in the supernatant of spleen cells from VL patients [27].

Of note, no significant correlations were found between the levels of IFN-γ and IL-10 detected between PHA and SLA stimulation, nor with the levels of arginase.

The results presented in the current study are in apparent contradiction with recent studies showing that IFN-γ is produced by whole blood cells from patients with active VL and that their IFN-γ levels were similar to those levels detected in cured patients [15]. Furthermore, our results also show that antigen specific IL-10 production in the WBA is not associated with active VL in Ethiopia, nor that it is produced by whole blood cells from cured patients in response to antigenic stimulation. Thus, our results clearly demonstrate that blood cells from patients with active VL are hyporesponsive, as activation with PHA resulted in significantly increased levels of IL-10 only in cured patients. Taken together our cytokine results show that blood cells from patients with active VL are hyporesponsive to both antigen-specific (as summarized in the review by Kumar et al. [7]) and polyclonal activation (as previously shown in [28–30]). The discrepancies between the results presented here and the above mentioned study might be explained by several factors:

1. The disease might be more severe in patients with active VL admitted to the *Leishmania* Research and Treatment Center in Gondar. In India, campaigns to raise awareness of VL have resulted in VL patients seeking early treatment and indeed, the duration of illness before treatment, defined as the number of weeks since the onset of symptoms, is significantly shorter in India than in Ethiopia: indeed, whereas the mean age of the patients appear to be similar in both studies (25.0±7.3 vs 29.5±17.6 years old), the duration of illness was considerably longer in the patients from Ethiopia as compared to India (56±40 days vs 37.31±35.29) [16]. This observation suggests that during the early stages of symptomatic VL, lymphocytes have maintained their capacity to respond to antigenic challenge, whereas a longer duration of illness without treatment is associated with immunosuppression. Furthermore, factors that might impact on disease severity such as BMI, anaemia and co-infections have not been compared in our respective studies in Bihar and in Gondar. Therefore we cannot exclude that other co-morbidities contributes to the profound immunosuppression and disease severity of patients with active VL patients in Ethiopia.
2. There might be genetic variation between the parasites. Indeed, there is an increased resistance of *L*. *donovani* to sodium stibogluconate in India, but not in Ethiopia [31]; on the other hand, patients in India respond successfully to treatment with one single dose AmBisome, but a similar clinical trial had to be terminated because of low efficacy in Gondar [32]. Furthermore East African *L*. *donovani* has been shown to be genetically different from the Indian *L*. *donovani* [33].
3. Differences in host genetic factors might also explain the discepancies between the Indian and the Ethiopian studies [34].

We cannot exclude that the low or undetected levels of IFN-γ might be due to the severe lymphopenia in VL patients (2.1±0.2 in VL patients vs 5.7±0.6 white blood cells (x10^3^); normal range = 4.5–10.5 white blood cells (x10^3^)), however, there was no correlation between the levels of IFN-γ in the supernatant of the WBA and the WBC counts (p = 0.1052, data not illustrated), suggesting that the low frequency of cells is unlikely to account for the observed levels of IFN-γ.

Despite the fact that we find little or no production of IFN-γ and IL-10 in the plasma harvested from the WBA at time of acute disease, these cytokines are clearly detected in the plasma of these patients directly *ex vivo* (summarized in [4]) demonstrating that these cytokines have been produced *in vivo*. Indeed, whereas the levels of these cytokines in the WBA in response to antigenic or polyclonal activation were below or barely above the levels of cytokines detected in the absence of stimulation, IFN-γ and IL-10 were detectable in the supernatant of the non-activated sample of whole blood cells (185.9±178.7 and 61.5±12.6 pg/ml, respectively) as well as in the plasma of these patients (123.0±27.5 and 88.9±12.5 pg/ml, respectively) (E. Adem, F. Tajebe, M. Getahun and P. Kropf, data not illustrated). This demonstrates that these cytokines are produced *in vivo*, but that whole blood cells cannot be induced to secrete these cytokines *in vitro* in response to activation. It is tempting to speculate that other cells, such as neutrophils, monocytes and spleen cells produce these cytokines.

In the current study, we show that whole blood cells from patients with active VL are hyporesponsive as no or low IFN-γ was released in response to activation. Since a recent study [16] showed that IFN-γ produced by antigen-specific CD4^+^ T cells contributes to the control of parasite replication in VL patients, it is possible that the lack of appropriate Th1 response might be responsible for the uncontrolled parasite replication in the patients in Gondar.

The use of the WBA has many advantages, such as being easy to perform, not requiring the use of sophisticated equipment and using only a small amount of blood. In addition, it is likely to contain all the factors necessary for cell activation and should mimic the *in vivo* conditions as closely as possible. The WBA provides a simple tool for determining cytokine profiles that may be useful laboratory predictors of early disease, aiding the evaluation of new interventions and offering insights into disease pathogenesis.
